# Testing phylogenetic signal with categorical traits and tree uncertainty

**DOI:** 10.1093/bioinformatics/btad433

**Published:** 2023-07-25

**Authors:** Diogo Ribeiro, Rui Borges, Ana Paula Rocha, Agostinho Antunes

**Affiliations:** CIIMAR/CIMAR, Interdisciplinary Centre of Marine and Environmental Research, University of Porto, Terminal de Cruzeiros do Porto de Leixões, 4450-208 Porto, Portugal; Department of Biology, Faculty of Sciences, University of Porto, Rua do Campo Alegre, 4169-007 Porto, Portugal; Institut für Populationsgenetik, Vetmeduni Vienna, 1210 Vienna, Austria; CMUP, Centre of Mathematics of the University of Porto, 4169-007 Porto, Portugal; Department of Mathematics, Faculty of Sciences, University of Porto, Rua do Campo Alegre, 4169-007 Porto, Portugal; CIIMAR/CIMAR, Interdisciplinary Centre of Marine and Environmental Research, University of Porto, Terminal de Cruzeiros do Porto de Leixões, 4450-208 Porto, Portugal; Department of Biology, Faculty of Sciences, University of Porto, Rua do Campo Alegre, 4169-007 Porto, Portugal

## Abstract

**Summary:**

The phylogenetic signal, frequently used to identify signatures of adaptive evolution or important associations between genes and phenotypes, measures the tendency for recently diverged species to resemble each other more than distantly related species. An example of such a measure is the *δ* statistic, which uses Shannon entropy to measure the degree of phylogenetic signal between a categorical trait and a phylogeny. In this study, we refined this statistic to account for tree uncertainty, resulting in more accurate assessments of phylogenetic associations. In addition, we provided a more accessible and computationally efficient implementation of the *δ* statistic that will facilitate its use by the evolutionary community.

**Availability and implementation:**

github.com/diogo-s-ribeiro/delta-statistic.

## 1 Introduction

The phylogenetic signal measures the tendency for species that have recently diverged to resemble each other more than those that are distantly related (Blomberg and Garland 2002). This signal between phenotypic traits and evolutionary histories helps us to understand the ways in which species evolve and become different.

Recently, we proposed the *δ* statistic, which is based on the concept of entropy from information theory. It exploits the uncertainty on the ancestral trait’s probability vectors, which can be inferred through maximum likelihood, Bayesian or Approximate Bayesian Computation (ABC) inference, to calculate the degree of phylogenetic signal between a categorical trait and a phylogeny (Borges *et al.* 2019). This statistic has been applied in various contexts, such as identifying homoplasic sites in SARS-CoV-2 sequences that can hinder phylogenetic reconstruction (Maio *et al.* 2020) or studying complex social traits, such as the parental care strategies of digger wasps (Field *et al.* 2020).

A limitation of this statistic is that it ignores common sources of error, and its previous implementation was not optimized for large-scale genomic studies. Here, we address these limitations by providing a new version of this statistic that accounts for tree uncertainty and developing a new Python implementation that will allow its use in large-scale genomic datasets, which are now commonplace in evolutionary biology. We also increased the accessibility and reproducibility of the *δ* statistic by facilitating its use by the evolutionary community with a web-application.

## 2 Implementation and usage

The previous *δ* statistic was implemented in R. Here, we converted it to the Python programming language, using the Numba library (Lam *et al.* 2015) for faster processing of data items. This new version utilizes the PastML package (Ishikawa *et al.* 2019), which automatically performs ancestral character reconstructions. The new implementation is on average 12.70 times faster for the standard use of 10 000 iterations (Supplementary Fig. S3). We expect this new implementation will allow applications with genome-scale data. We have also provided a web application that includes a tutorial explaining the usage of the *δ* statistic and detailed information about the input and output data. This webserver takes on average 10 s per tree (Supplementary Fig. S2) and can be used for smaller-scale applications or for teaching purposes.

## 3 Application

The previous *δ* statistic of phylogenetic signal assumed that the given tree is correct (Borges *et al.* 2019), ignoring possible and likely uncertainties on the topology and branch lengths. To test the impact of tree uncertainty, we used mammalian data retrieved from OrthoMAM (Scornavacca *et al.* 2019). Our dataset consisted of 1000 protein-coding sequence alignments for 30 mammals. To avoid possible biases due to gene length (i.e. longer genes are expected to estimate more accurate trees), the alignments were trimmed to 1000 base pairs. Our categorical traits consisted of a 2-class (presence and absence of meat in primary diet) and a 3-class (carnivorous, omnivorous, and herbivorous) phenotypes that were defined for the 30 mammalian species based on existing literature (Yahnke *et al.* 2013) (Supplementary Table S1 and Supplementary Fig. S5). Gene tree estimation was conducted with the Bayesian software RevBayes (Höhna *et al.* 2016) (further details in Supplementary Text).

**Figure 1.**
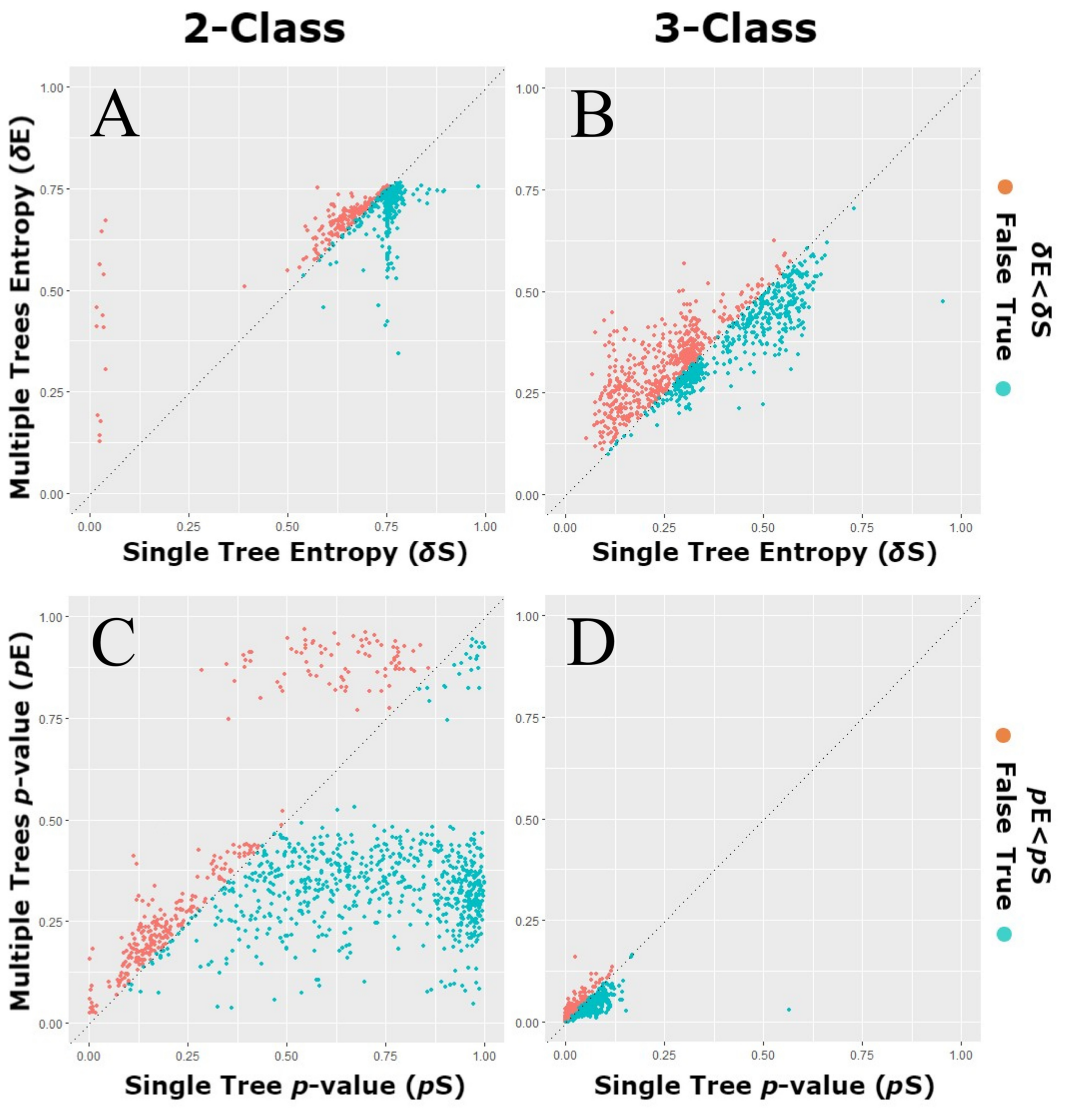
Impact of tree uncertainty on the *δ* statistic. (**A**, **B**) Estimated entropies for the standard (*δ*_S_) and new (*δ*_E_) *δ* statistic in a categorical trait of 2 and 3 classes. (**C**, **D**) Probability *P* of a phylogenetic association for the standard (*p*S) and new method (*p*E)

To evaluate the impact of tree uncertainty on the phylogenetic signal, we compared the *δ* statistic calculated using the previous method (*δ*_S_) and the extended method proposed here (*δ*_E_) when accounting for multiple phylogenetic trees. We used 1000 randomly sampled trees from the posterior distribution to average *δ*_E_. However, we found that *δ*_E_ converges after 840 and 580 trees for the 2- and 3-character traits across all genes (Supplementary Fig. S7). We observed that the two entropies do not have a one-to-one relationship (Fig. 1), with higher *δ*_S_ values having corresponding smaller *δ*_E_ values and vice versa. These discrepancies are related to the overall support of the phylogeny. While *δ*_S_ and *δ*_E_ are similar for trees that have higher posterior clade probabilities, they differ for trees that have overall low support. This result is expected, as for low-supported trees, the maximum a posteriori tree (MAP) might not represent a reasonable gene history. Accounting for distinct posterior-sampled trees thus seems to help recover phylogenetic signal associated with clades that are not present in the MAP.

In addition to the entropy comparison, we also computed the probability, *P*, of finding the empirical entropy value under the standard and new method in their null distributions (Δ_S_ and Δ_E_, respectively) (Supplementary Table S3), obtained by shuffling the trait vector at the tree tips (Equation 1).


(1) $$p_{S}=P(\Delta_{S}<\delta_{S})\;\;\;\text{and}\;\;\;p_{E}=P(\Delta_{E}<\delta_{E}).$$

This probability serves as a proxy for the *P*-value. We observed that the values of *P* under the new method are on average 1.37 times smaller (2-class: 1.64 times; 3-class: 1.10 times) than in the standard method (Fig. 1 and Supplementary Table S2). This shows that the new method is more likely to identify phylogenetic associations and indicates that accounting for tree uncertainty captures evolutionary signal.

## 4 Conclusions

The revised *δ* statistic provides a more accurate discovery of phylogenetic associations, thus delivering a more precise characterization of the genetic players involved in species adaptation. Moreover, our faster and more accessible *δ* statistic will facilitate its automated use in modern genomic applications, including phylogenetic pipelines with large genomic datasets, or in the classroom for teaching purposes.

## Supplementary Material

btad433_Supplementary_Data

## Data Availability

The data underlying this article are available at Zenodo (https://doi.org/10.5281/zenodo.7541548).
