# Supplementary material for: Testing phylogenetic signal with categorical traits and tree uncertainty
Source: Bioinformatics. 2023 Jul 25;39(7):btad433. doi: 10.1093/bioinformatics/btad433 (PMC10374490; doi:10.1093/bioinformatics/btad433)
Supplement: btad433_Supplementary_Data [file btad433_supplementary_data.zip › Bioinformatics(AN)-Delta_statistic_Supplementary.pdf]

Supplementary material of *Testing phylogenetic signal with categorical traits and tree uncertainty* by Diogo Ribeiro, Rui Borges, Ana Paula Rocha and Agostinho Antunes

Supplementary text

Several methods have been developed to measure the phylogenetic signal that use a multitude of approaches and statistics. They frequently address similar ecological and evolutionary questions (e.g., properties of species, their habitat and phylogenetic relationships) (Simmons and Ochoterena, 2000; Pearman *et al.*, 2008; Kamilar and Cooper, 2013), while focusing and quantifying different aspects of the phylogenetic signal (Münkemüller *et al.*, 2012). The majority of indexes of phylogenetic signal were developed for continuous traits (Moran, 1950; Abouheif, 1999; Pagel, 1999; Blomberg *et al.*, 2003). Those for discrete traits lag far behind, as calculating variances and covariances with categorial data is challenging (Fritz *et al.*, 2010).

Phylogenetic analyses

Gene tree estimation was conducted with the Bayesian software `RevBayes` Höhna *et al.* (2016), under the general time reversible (GTR+Gamma+I) model (Tavaré, 1986) with a uniform Dirichlet prior for the nucleotide composition and their exchangeabilities (Heath *et al.*, 2012). The Markov chain Monte Carlo (MCMC) algorithm was employed with two independent chains and one million generations and saving only every 10th generation (to decrease the autocorrelation between samples). Overall, 15% of the samples were burned-in (Kruschke, 2015). Convergence and mixing were assessed using visual inspection in the `Tracer` package (Rambaut *et al.*, 2018) (figure S3) and tested for numerical convergence using the Gelman-Rubin index (Gelman and Rubin, 1992) in the python package `ChainConsumer` (Hinton, 2016).

From the resulting sampled trees, we created an exhaustive code that uses the `ace` package in R, which compares each topology and determines their frequency. A sample of 1000 trees and their sample frequency was created for each gene.

Computation

For ancestral character reconstruction, our approach primarily relies on the PastML package (Ishikawa *et al.*, 2019), which offers a direct implementation in Python. However, for those who prefer alternative tools, the ape package Paradis and Schliep (2018) can also be utilized by integrating it with r2Py. The δ statistic code was converted to the Python programming language to take advantage of its straightforward code, and the `NumPy` library (Harris *et al.*, 2020) made the code more compact. Additionally, Python has a fast growth, currently being the most popular coding language, as measured by both the TIOBE (Jansen, 2000) and PYPL (Carbonnelle, 2018) indexes, having grown the most in the last five years.

Having access to an easy-to-use interface is usefull for teaching purposes or for smaller-scale applications. As such, a δ statistic web application was created using the `Django` framework (Django Software Foundation, 2019). Overall, it was created using Python, CSS, JavaScript, and HTML.

Results

The null distribution is the base for hypothesis testing. To obtain it we randomly assigned character traits to the tree tips and then calculated the resulting entropy. We did this for the standard method and for the the multiple trees. We compare the resulting null-distributions in terms of their 5th, 25th, 50th, 75th and 95th percentiles. The expections is that if these statistics are similar, then the null distributions generated by these two methods are similar (figure S5).

Since the quantiles show very similar values across methods, we conclude that the null distribution might not be responsible for differences in the detected target between the methods and, instead, this variation might be because of the way we calculate the δ statistic. As such, both methods can be used to calculate the null-hypothesis, and this would result in similar values. Additionally, when analyzing their distribution, their results also seem to be relatively congruent, not changing much except the 5th percentile that, in the 2-class trait, seems to vary considerably among methods but a trend was not observed.

In our analyses we have used 1000 trees from the posterior distribution to establish an average entropy. We used this data to calculate a moving average of the δ statistics as we increase the number of sampled trees from 1 to 1000. We observed that the measures of the δ statistic converge well as the number of sampled trees increased and that a total of 840 and 580 random trees for the 2-Class and 3-Class scenarios, respectively, would be sufficient to estimate δ with variability less than 5% from the entire range of markers that we have used. We conclude that our 1000 is conservative and may be applied to the generality of cases. Alternatively, for a less conservative option, we observed that around 200 random trees would be sufficient for 95% of the markers to converge (figure S6). Furthermore, we observed that the time for this problem is linear, with our python implementation taking 0.342 seconds per tree for the standard use of 10000 iterations (figure S2).

Finaly, we have also tested the gene evolutionary rate. Genes evolve differently by incoporating substitutions at different rates; this is expected to have an impact on the estimated entropies. As such, an uniform random sample of 1000 was made when selecting the markers. However, no correlation was found between the evolutionary rate and the two methods we employed to measure the entropies. This might indicate that, independently from its values, the new method does not distort the results.

SUPPLEMENTARY REFERENCES

[S1]Abouheif, E. (1999). A method for testing the assumption of phylogenetic independence in comparative data. *Evolutionary Ecology Research*, **1**, 895–909.

[S2]Blomberg, S. P. *et al.* (2003). Testing for phylogenetic signal in comparative data: Behavioral traits are more labile. *Evolution*, **57**, 717–745.

[S3]Carbonnelle, P. (2018). Pypl popularity of programming language index. Accessed: 2022-09-30.

[S4]Django Software Foundation (2019). Django.

[S5]Fritz, S. *et al.* (2010). Selectivity in mammalian extinction risk and threat types: a new measure of phylogenetic signal strength in binary traits. *Conservation Biology*, **24**, 1042–1051.

[S6]Gelman, A. and Rubin, D. (1992). Inference from iterative simulation using multiple sequences. *Statistical Science*, **7**, 457–472.

[S7]Harris, C. R. *et al.* (2020). Array programming with NumPy. *Nature*, **585**(7825), 357–362.

[S8]Heath, T. *et al.* (2012). A dirichlet process prior for estimating lineage-specific substitution rates. *Molecular Biology and Evolution*, **29**, 939–955.

[S9]Hinton, S. R. (2016). ChainConsumer. *The Journal of Open Source Software*, **1**, 00045.

[S10]Höhna, S. *et al.* (2016). RevBayes: Bayesian Phylogenetic Inference Using Graphical Models and an Interactive Model-Specification Language. *Systematic Biology*, **65**(4), 726–736.

[S11]Ishikawa, S. A. *et al.* (2019). A fast likelihood method to reconstruct and visualize ancestral scenarios. *Molecular Biology and Evolution*, **36**, 2069–2085.

[S12]Jansen, P. (2000). Tiobe index | tiobe - the software quality company. Accessed: 2022-09-30.

[S13]Kamilar, J. M. and Cooper, N. (2013). Phylogenetic signal in primate behaviour, ecology and life history. *Philosophical Transactions of the Royal Society B: Biological Sciences*, **368**.

[S14]Kruschke, J. K. (2015). Markov chain monte carlo. *Doing Bayesian Data Analysis*, pages 143–191.

[S15]Moran, P. A. P. (1950). Notes on continuous stochastic phenomena. *Biometrika*, **37**, 17–23.

[S16]Münkemüller, T. *et al.* (2012). How to measure and test phylogenetic signal. *Methods in Ecology and Evolution*, **3**(4), 743–756.

[S17]Pagel, M. (1999). Inferring the historical patterns of biological evolution. *Nature*, **401**.

[S18]Paradis, E. and Schliep, K. (2018). ape 5.0: an environment for modern phylogenetics and evolutionary analyses in R. *Bioinformatics*, **35**(3), 526–528.

[S19]Pearman, P. B. *et al.* (2008). Niche dynamics in space and time. *Trends in Ecology & Evolution*, **23**, 149–158.

[S20]Rambaut, A. *et al.* (2018). Posterior summarization in bayesian phylogenetics using tracer 1.7. *Systematic Biology*, **67**, 901–904.

[S21]Simmons, M. and Ochoterena, H. (2000). Gaps as characters in sequence-based phylogenetic analyses. *JSTOR*.

[S22]Tavaré, S. (1986). Some Probabilistic and Statistical Problems in the Analysis of DNA Sequences. *Lectures on Mathematics in the Life Sciences*, **17**, 57–86.

Supplementary tables

Specie		Family	2-Class	3-Class
Ailuropoda	melanoleuca	Ursidae	No	Herbivorous
Bos	mutus	Bovidae	No	Herbivorous
Camelus	bactrianus	Camelidae	Yes	Omnivorous
Canis	lupus	Canidae	Yes	Carnivorous
Capra	aegagrus	Bovidae	No	Herbivorous
Castor	canadensis	Castoridae	No	Herbivorous
Cebus	capucinus	Cebidae	Yes	Omnivorous
Ceratotherium	simum	Rhinocerotidae	No	Herbivorous
Chlorocebus	sabaeus	Cercopithecidae	No	Herbivorous
Colobus	angolensis	Cercopithecidae	No	Herbivorous
Cricetulus	griseus	Cricetidae	Yes	Omnivorous
Enhydra	lutris	Mustelidae	Yes	Carnivorous
Eptesicus	fuscus	Vespertilionidae	Yes	Carnivorous
Equus	africanus	Equidae	No	Herbivorous
Homo	sapiens	Hominidae	Yes	Omnivorous
Macaca	fascicularis	Cercopithecidae	Yes	Omnivorous
Mus	musculus	Muridae	Yes	Omnivorous
Nomascus	leucogenys	Hylobatidae	No	Herbivorous
Odobenus	rosmarus	Odobenidae	Yes	Carnivorous
Odocoileus	virginianus	Cervidae	No	Herbivorous
Orcinus	orca	Delphinidae	Yes	Carnivorous
Orycteropus	afer	Orycteropodidae	Yes	Carnivorous
Otolemur	garnettii	Galagidae	Yes	Omnivorous
Panthera	pardus	Felidae	Yes	Carnivorous
Papio	anubis	Cercopithecidae	Yes	Omnivorous
Physeter	catodon	Physeteridae	Yes	Carnivorous
Rhinolophus	sinicus	Rhinolophidae	Yes	Carnivorous
Sus	scrofa	Suidae	Yes	Omnivorous
Trichechus	manatus	Trichechidae	No	Herbivorous
Tupaia	belangeri	Tupaiidae	Yes	Omnivorous

Table 1. Specie’s scientific name, family and respective trait information that was analyzed in this project.

	2-Class		3-Class	
**p-value**	**pS $\geq$ 5%**	**pS < 5%**	**pS $\geq$ 5%**	**pS < 5%**
**pE $\geq$ 5%**	984	5	95	20
**pE < 5%**	3	8	104	781

Table 2. Confusion table of a phylogenetic association for the standard (p_S) and new method (p_E).

	2-Class				3-Class			
	δ_S	δ_E	**pS**	**pE**	δ_S	δ_E	**pS**	**pE**
**Minimum**	0.016	0.128	0.001	0.024	0.054	0.098	0.000	0.000
**Lower-hinge**	0.748	0.702	0.359	0.241	0.236	0.272	0.007	0.012
**Median**	0.752	0.734	0.626	0.329	0.317	0.333	0.016	0.019
**Upper-hinge**	0.752	0.747	0.894	0.408	0.457	0.421	0.036	0.032
**Maximum**	0.911	0.765	1.000	0.968	0.953	0.702	0.564	0.165
**Mean**	0.727	0.713	0.598	0.366	0.336	0.345	0.028	0.026
**Standard deviation**	0.094	0.063	0.298	0.206	0.147	0.103	0.035	0.022

Table 3. Values of multiple statistic measures for δ_S, δ_E, pS and pE.

Supplementary figures

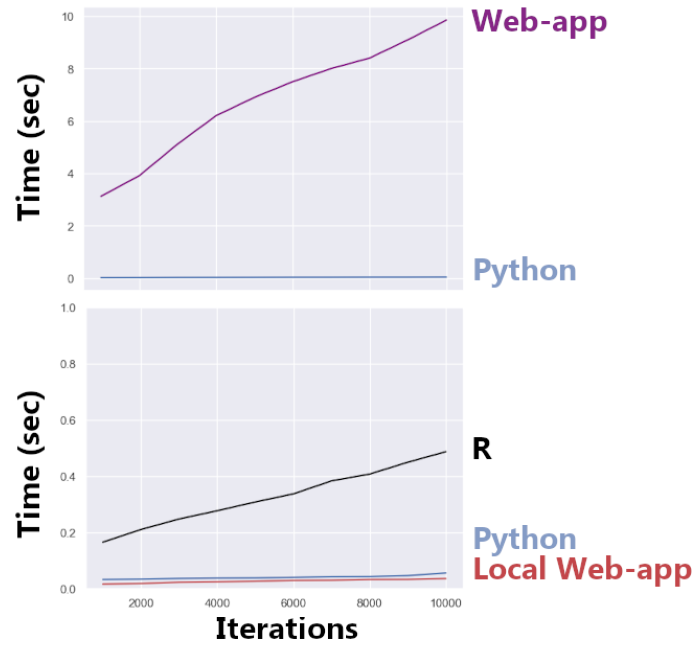

Fig. 1. Time taken by the various implementations of δ statistic in each tree for 1000-10000 iterations.

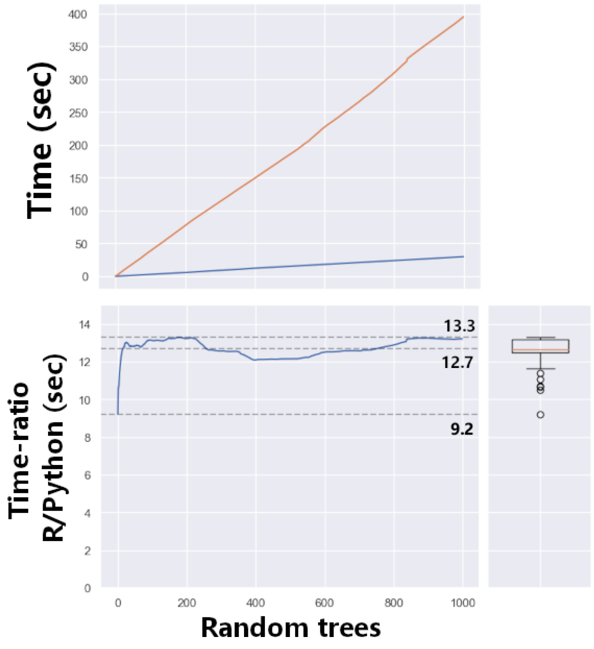

Fig. 2. Time taken by the R and Python code for the standard use of 10000 iterations with 1-1000 trees and their respective ratio.

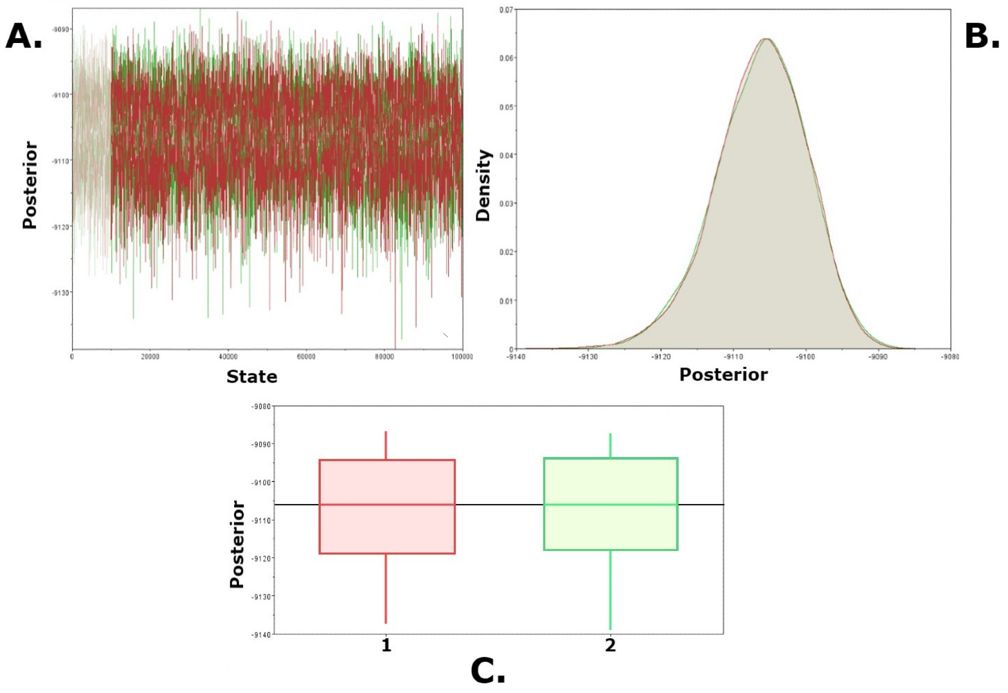

Fig. 3. Visual examination of the CTU2 marker in the Tracer package by analyzing both runs. A. trace plots, B. density plots and C. box-plots.

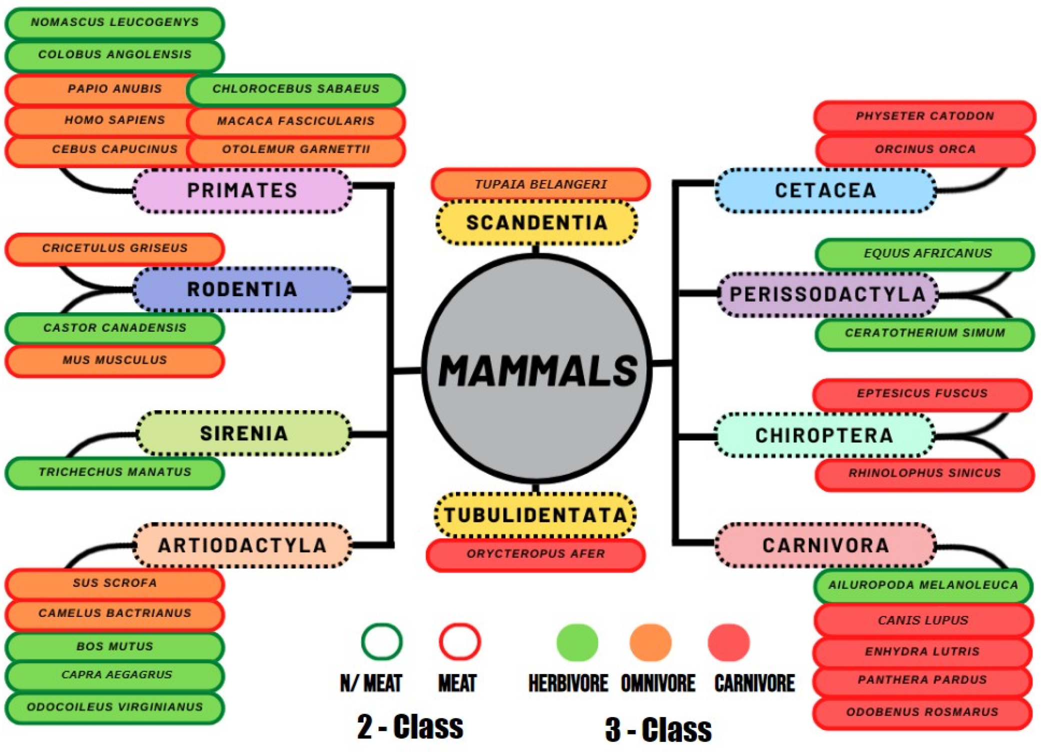

Fig. 4. Species (and respective order) that were analyzed in this project.

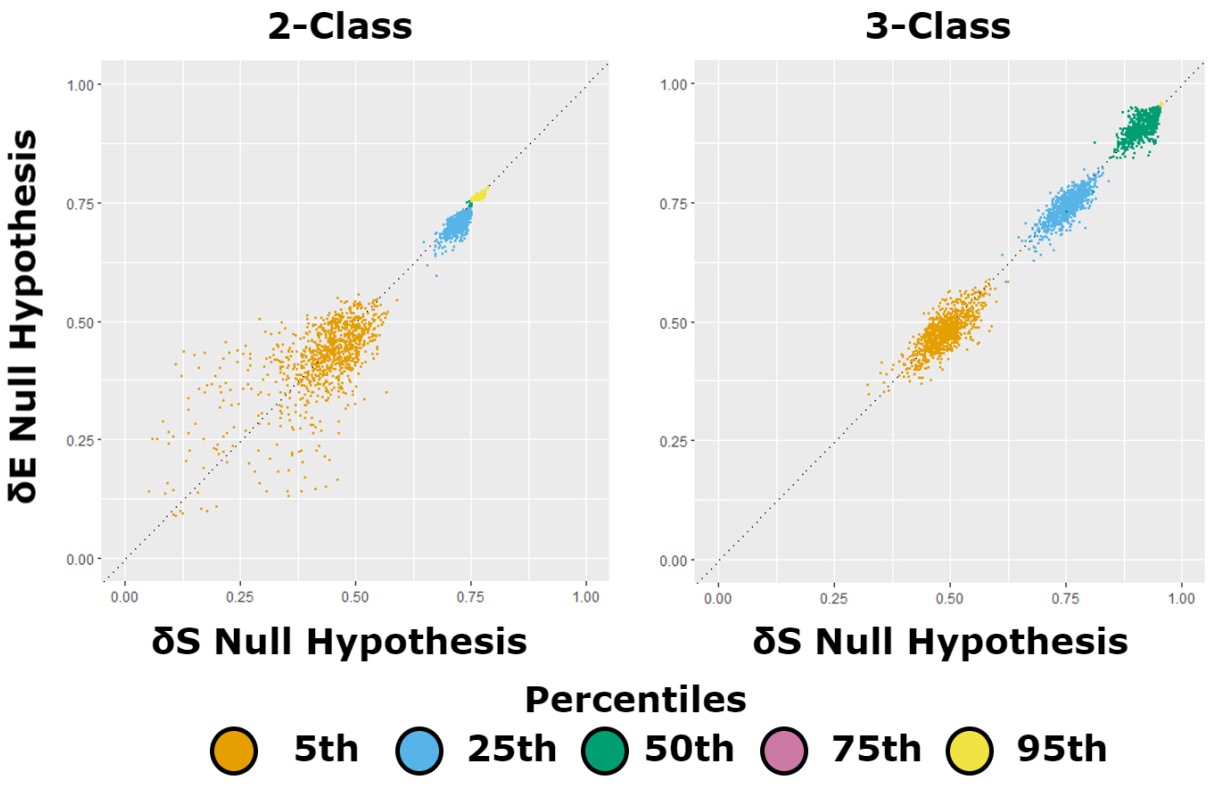

Fig. 5. Distribution of the null hypothesis results in the different methods and k-class traits, when analyzing the sample of 1000 markers.

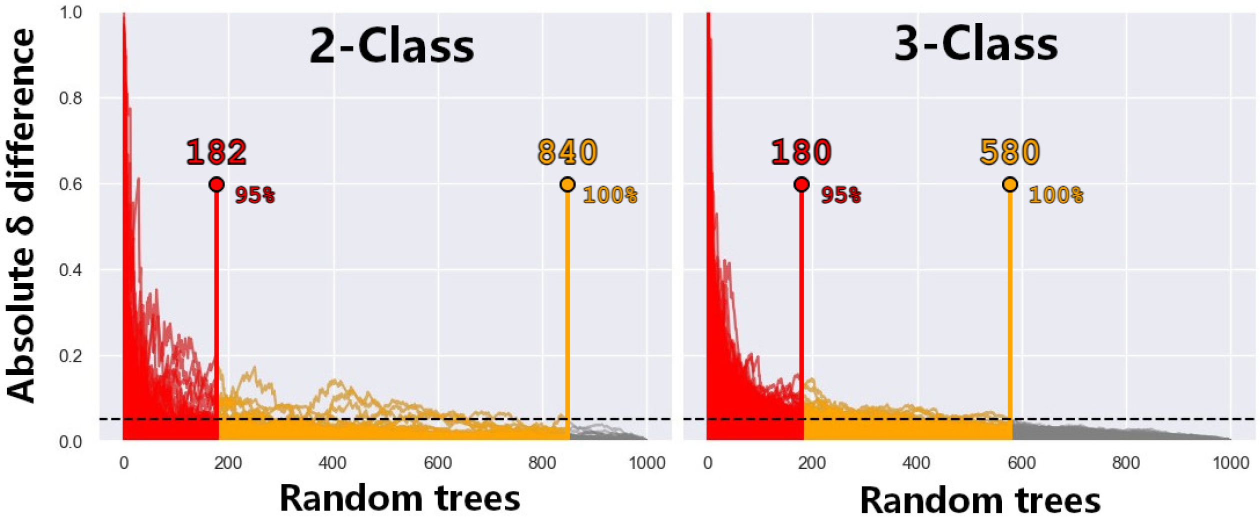

Fig. 6. Convergence of δ_S-values as the number of randomly sampled trees increases. The red threshold indicates the minimum number of sample trees required to achieve an absolute error below 5% in 95% of the studied genes, while the orange threshold represents this criterion for all 1000 genes.
